# Effect of Dwarfing Gene *Ddw1* on Height and Agronomic Traits in Spring Triticale in Greenhouse and Field Experiments in a Non-Black Earth Region of Russia

**DOI:** 10.3390/plants8050131

**Published:** 2019-05-16

**Authors:** Pavel Kroupin, Anastasiya Chernook, Gennady Karlov, Alexander Soloviev, Mikhail Divashuk

**Affiliations:** 1 Laboratory of Applied Genomics and Crop Breeding, All-Russia Research Institute of Agricultural Biotechnology, Timiryazevskaya str. 42, Moscow 127550, Russia; irbis-sibri@yandex.ru (A.C.); karlov@iab.ac.ru (G.K.); 2 Centre for Molecular Biotechnology, Russian State Agrarian University – Moscow Timiryazev Agricultural Academy, Timiryazevskaya street, 49, Moscow 127550, Russia; 3 Laboratory of Marker-Assisted and Genomic Selection of Plants, All-Russia Research Institute of Agricultural Biotechnology, Timiryazevskaya str. 42, Moscow 127550, Russia; soloviev@iab.ac.ru; 4 Department of Distant Hybridization, N.V. Tsitsin Main Botanical Garden of Russian Academy of Sciences, Botanicheskaya str., 4, Moscow 127276, Russia

**Keywords:** triticale, breeding, germplasm, harvest index, dwarfing genes, agronomic traits, *Ddw1*, Hl, wheat, rye, wide hybridization, PCR, fragment analysis

## Abstract

Triticale is a relatively new crop which still possesses serious drawbacks that can be significantly improved by breeding. The dwarfing genes proved to be very useful in the development of new lodging resistant and productive cultivars of winter triticale. The aim of our research was to assess the effect of the *Ddw1* dwarfing gene from rye on the agronomic valuable traits in spring triticale. The *Ddw1* effect was studied in the greenhouse experiment in segregating the F_2:3_ population and in the field of F_3:4_ and F_4:5_ families derived from crossing winter triticale ‘Hongor’ (*Ddw1Ddw1*) and spring triticale ‘Dublet’ (*ddw1ddw1*). As a result, in all three generations, a strong decrease in plant height was demonstrated that was accompanied by a decrease in grain weight per spike and 1000-grain weight. In field experiments, a decrease in spike length and increase in spike density and delay in flowering and heading were observed. As a result of decrease in culm vegetative weight due to *Ddw1*, the harvest index measured in F_4:5_ increased. The spike fertility and number of grains were not affected by *Ddw1*. The comparison of *Ddw1* in rye, winter, and spring triticale, and the possible role of *Ddw1* in improving spring triticale are discussed.

## 1. Introduction

Triticale is a relatively recently developed crop. However, triticale gained a solid position in production of grain. In the recent five years in the Russian Federation, the total area of triticale averaged 225,000 hectares, similar to the area sown by rice, and the total crop yield was on average 584,000 tons, thus constituting 7% of the whole crop yield of Eastern Europe [1,2]. The main use of triticale in Russia involves the production of feeds for animal husbandry and the manufacture of raw materials for production of alcohol for the fermentation industry. 

The development of cultivars adapted to various local agricultural and climate conditions is required for sustainable agriculture. In Russia, current triticale breeding efforts include increase in yield and resistance to biotic and abiotic stress with particular emphasis on resistance to lodging [3,4,5,6]. Development of the dwarf and semidwarf varieties of triticale is the most reliable solution for the lodging problem. Results of the study performed using a collection of 199 winter and 2 spring triticale varieties indicated that height selection during the past 30 years can lower the height of triticale varieties at a rate of 0.38 cm per year on average; this breeding significantly enhanced resistance to lodging [7]. It is known that shorter plants are usually more resistant to lodging [4,8]. Height of the triticale plants is positively correlated with the biomass and negatively correlated with the yield index [9,10]. Various studies have obtained variable results on the effect of the height of the plants on certain structural elements of the yield, including spike length, grain number per spike, spikelet number per spike, 1000-grain weight, and grain yield; these parameters are apparently determined by specific genotypes and experimental conditions [7,11,12,13]. 

The height of triticale can be lowered via dwarfing genes of wheat and rye. In rye, plant height is determined by numerous genetic factors [14,15]. At present, 14 dwarfing rye genes have been identified. The dominant *HI* gene, subsequently renamed as *Ddw1*, is the most valuable gene for breeding; the gene has been discovered in an EM-1 rye mutant [16,17]. The dominant gene *Ddw1* reduces plant height in diploid and tetraploid rye by 40% and 55%, respectively [16,18]. The effects of the *Ddw1* gene in rye are pleiotropic and are manifested as the shortening of the internodes of the culm, enhanced tillering of the pants, increased size of the leaves and spikes, and increase in the number of spikes and seeds in a spike [16].

In Russia, a number of agriculturally valuable triticale varieties and lines (winter and spring-and-winter lines) carrying the dwarfing *Ddw1* gene were developed. The lines had enhanced productivity and bread-making quality and were developed in Dagestan experimental station of N.I. Vavilov Institute of Plant Industry (VIR) (PRAG 199, PRAG 184, and PRAG 531) and in Krasnodar Research Institute of Agriculture, named after P. Lukyanenko (‘Valentin 90′, ‘Hongor’, ‘Avangard’, and ‘Mudrets’) [19,20,21,22]. In Poland and Romania, a number of dwarf or semidwarf winter triticale varieties have been developed with the use of *Ddw1* as well [23,24,25]. Alheit et al. [15] and Kalih et al. [26] have identified a QTL at the distal region of chromosome 5R of hexaploid triticale associated with plant height and the authors suggested that the region might correspond to the dominant dwarfing allele *Ddw1*.

Introgression of *Ddw1* into the primary octaploid winter triticale varieties [27] lowered the height by 38.4% or by 23.5 cm (24.5%) [28]; in the hexaploid winter triticale, *Ddw1* lowered the plant height by 20% (20 cm) on average [26]; in the secondary triticale obtained by crossing of octaploid and hexaploid triticale, the height was lowered by 20% (20 cm) on average [23]. Similar to rye, the *Ddw1* gene has pleiotropic effects in the primary octaploid triticale, reducing leaf length and enhancing productive tillering by 13% [27]. In the hexaploid triticale with *Ddw1*, the yield of grain was decreased by 0.43 tons/ha while reducing resistance to Fusarium head blight and delaying flowering [26]. The effect of *Ddw1* on valuable agronomic traits in the spring hexaploid triticale has not been reported in the literature.

Russia has a large territory with contrasting climatic conditions. The Non-Black Earth region (Nechernozemye), with its sod-podzol soils and excessive rainfalls, is characterized as an unfavorable agricultural zone. It accounts for 1/5 of all the agricultural lands of Russia, with 1/3 of its population living there. Therefore, it requires its own competitive and sustainable agriculture with crop varieties adapted to the local environment. Triticale is very promising for the Non-Black Earth regions of Russia due to its sufficient plasticity and its resistance to unfavorable growth conditions and fungal diseases. The problem of lodging is urgent for Russia since strong winds and heavy rainfalls are very usual during harvesting in the Non-Black Earth region.

We aimed to assess the effect of *Ddw1* on the plant height and on traits of agronomic importance in the spring hexaploid triticale in the F_2:3_ hybrid population in greenhouse experiments, and in the F_3:4_ and F_4:5_ families in the field in Non-Black Earth region of Russian Federation by crossing the ‘Hongor’ (*Ddw1Ddw1*) and ‘Dublet’ (*ddw1ddw1*) cultivars.

## 2. Results

**Figure 1:**
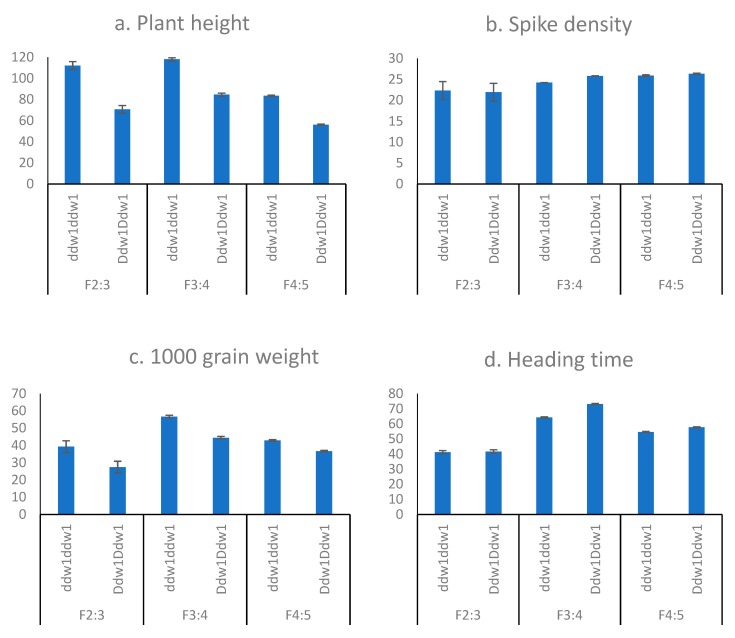
Agronomic traits in spring triticale population ‘Hongor’ × ’Dublet’ F_2:3_ and families F_3:4_ and F_4:5_ with *ddw1ddw1* and *Ddw1Ddw1* genotypes: (**a**) plant height (cm); (**b**) spike density; (**c**) 1000 grain weight (g); (**d**) heading time (days). Bars indicate LSD_0.05_.

The effect of *Ddw1* on height and other agronomic traits of the hexaploid spring triticale have not been described in the literature. Although *Ddw1* has been introgressed into the genome of winter triticale, its effects may depend on the rate of plant growth and conditions of formation and development of various organs. Thus, we have assessed the effect of *Ddw1* on height and productivity elements (Figure 1).

### 2.1. Plant Height

The presence of *Ddw1* in F_2:3_ significantly lowers the height by 41.5 cm (37.0%); in F_3:4_, height was lowered by 33.4 cm (28.4%); and in F_4:5_, height was lowered by 27.2 cm (32.6%). Heterozygous plants were detected in F_2:3_ due to crossing of the F_1_ generation, and their mean height was significantly different from the height of the homozygous *Ddw1Ddw1* plants indicating that *Ddw1* is a partially dominant allele or that there is an effect of the gene dose (Figure 1a, Table S1).

*Ddw1* decreased the number of internodes in the greenhouse F_2:3_ experiment by 0.3 (5.8%) and in the field F_4:5_ experiment by 0.1 (1.9%). Correlation between the number of internodes and height was of medium strength in the vegetation experiment and of medium-low strength in the F_4:5_ field experiment (Table 1).

**Figure 2:**
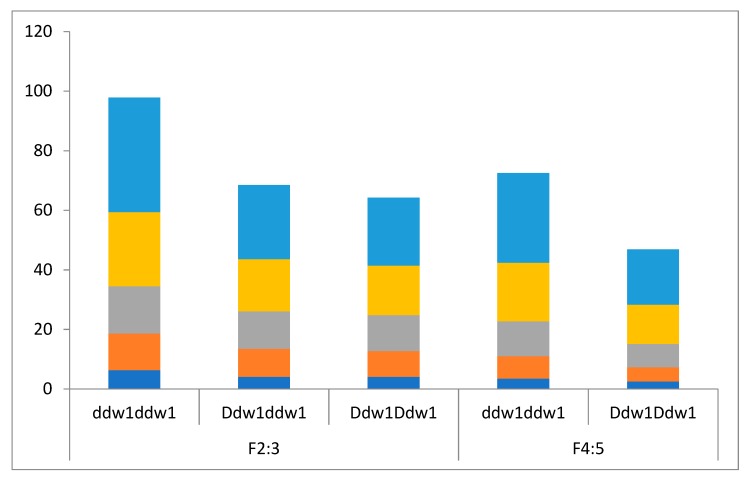
Chart of internode length of the triticale plants in the greenhouse F_2:3_ and field F_4:5_ experiments. The colors of the charts stand for the following internodes: light blue, peduncle; violet, 2nd upper internode; green, 3rd upper internode; red, 2nd lower internode; and dark blue, 1st lower internode. Horizontal axis: 0, *ddw1ddw1* genotype; 1, *Ddw1ddw1* genotype; and 2, *Ddw1Ddw1* genotype.

In the F_2:3_ greenhouse and F_4:5_ field experiments, a decrease in the internode length was significant in all internodes and varied from 23.9 to 40.6% in F_2:3_ and from 28.6 to 38.2% in F_4:5_ (Figure 2).

In the greenhouse F_2:3_ experiment, an absolute decrease in the internode length in the case of *Ddw1* was the highest in the peduncle internode (15.6 cm) and in the second upper internode (8.3 cm); in the field F_4:5_ experiment, similar to the greenhouse experiment, the decrease was the highest in the peduncle internode (11.5 cm) and in the second upper internode (6.5 cm). In the greenhouse F_2:3_ experiment, the relative decrease was the highest in the peduncle internode (40.6%) and in the first lower (the lowest) internode (34.9%); in the field F_4:5_ experiment, the decrease was the highest in the peduncle internode (38.2%) and in the second lower internode (36.0%). In the greenhouse F_2:3_ experiment, a decrease in length in the peduncle and in the first lower internodes was 38% and 20% of total plant height decrease, respectively; in the field F_4:5_ experiment, a decrease in length in the peduncle and in the first lower internodes was 42% and 24%, respectively (Table S1). Association between the plant height and the *Ddw1Ddw1* genotype was the strongest in the peduncle internode in the greenhouse F_2:3_ and field F_4_:_5_ experiments (Table 1). A decrease in the length of the internodes induced by *Ddw1* had similar ratios of the lengths of the internodes compared to those of the homozygotes and heterozygotes of *ddw1* in F_2:3_ and F_4:5_ (Table 2).

Thus, a decrease in height induced by *Ddw1* in the plants of spring triticale in our experiments can be explained by a decrease in the length of the peduncle and upper internodes, and is not due to a decrease in the number of internodes.

### 2.2. Spike Architecture and Productivity

A considerable decrease in spike length was induced by *Ddw1* in the greenhouse F_2:3_ experiment, although this decrease was not statistically significant. In the F_3:4_ field experiment, no significant changes were observed in this trait, while in F_4:5_, the spike length was decreased by 0.24 cm (2.2%) (Table S1). 

Spikelet number per spike was significantly changed only in the field F_3:4_ experiment increasing by 1.5 spikelets per spike (5.9%). *Ddw1* increased spike density by 1.6 (6.5%) in F_3:4_ and by 0.4 (1.7%) in F_4:5_ (Figure 1b, Table S1). Thus, an increase in the spike density due to *Ddw1* in the field experiments in F_3:4_ is because of increase in spikelet number per spike, while in F_4:5_ it is owing to a decrease in spike length.

Neither grain number per spike nor grain number per spikelet were affected significantly by *Ddw1* presence (Table S1). Thus, spike fertility is not influenced by *Ddw1* in the greenhouse and two-year field experiments.

The dwarfing *Ddw1* gene induced a decrease in grain weight per spike in all three generations (F_2:3_, F_3:4_, and F_4:5_) by 0.7 g (35%) in F_2:3_, by 0.7 g (18.8%) in F_3:4_, and by 0.3 g (14.3%) in F_4:5_. Moreover, *Ddw1* induced a decrease in 1000-grain weight in all three experiments by 11.8 g (30.1%) in F_2:3_, by 12.3 g (21.8%) in F_3:4_, and by 6.3 g (14.7%) in F_4:5_ (Figure 1d, Table S1). The number of grains was not influenced by *Ddw1*, indicating that the size of the grains decreased. 

*Ddw1* did not influence productive tillering in all experiments (Table S1).

### 2.3. Flowering and Heading

*Ddw1* did not significantly influence the time of flowering and heading in the F_2:3_ greenhouse experiment. However, in the field experiment, *Ddw1* induced a delay of 9 and 3 days in heading in the case of F_3:4_ and F_4:5_, respectively. Flowering was delayed by 4 days on average in F_3:4_ and F_4:5_ (Figure 1d, Table S1).

### 2.4. Harvest Index

Harvest index considers two parameters, including grain weight and vegetative weight of the harvested plant. In the field F_4:5_ experiment, we measured the weight of the main spike culm and the weight of the unthreshed main stem. In the field F_4:5_ experiment, in *Ddw1Ddw1* plants the harvest index was 5.4% higher than in *ddw1ddw1* (5.4%). In F _4:5_, *Ddw1* induced a significant decrease in the vegetative weight of the main spike from 2.8 to 2.4 g (14.6%) and a decrease in the vegetative weight of the main stem from 1.0 g to 0.6 g (38.5%) (Table 3). Thus, the overall harvest index is significantly increased despite a decrease in the grain weight of the main spike and the vegetative weight of the spike and culm.

### 2.5. Impact of Gene and Family

Regression analysis was used every year to assess the impact of family and the impact of the dwarfing gene towards certain features (Table 4). 

PH, plant height (cm); SL, spike length (cm); SNS, spikelet number per spike; SD, spike density; GWS, grain weight per spike (g); GNS, grain number per spike; GNSpl, grain weight per spikelet (g); 1000-grain weight (TGW, g); NFT, number of fertile tillers; NI, number of internodes; HD, heading date; and FD, flowering date.

The results listed in Table 4 indicate that in the case of F_3:4_, the contribution of family to the genotypic variability of the traits is statistically significant in height (40%), weight of 1000 grains (29%), time of flowering (up to 17%) and heading (20%), and spike density (19%). In F_4:5_, the impact of family on genotypic variability is significantly lower and ranges from 1% to 4%. Additionally, the data describe the strength of associations between the alleles of the *Ddw1* gene and phenotypic features. For example, in F_2:3_, features related to the plant height and weight of 1000 grains were significantly correlated with a high correlation coefficient, and a medium correlation coefficient was observed in the case of associations with spike density, weight of grains of the main spike, and time of flowering and heading. In F_4:5_, only a single strong association of the *Ddw1* gene with plant height was detected. Association with spike density was decreased down to the weak level, and associations with grain weight of the main spike and time of heading and flowering remained constant, whereas associations of the *Ddw1* gene with the 1000-grain weight decreased down to the medium level.

## 3. Discussion

Decrease in the height of the spring triticale observed in our study was more pronounced than that obtained in the winter triticale. For example, in our experiments, the decrease varied from 27.2 cm to 41.5 cm (28.4–37.0%), whereas in winter triticale, the decrease was 20 cm (20%) [26,28]. Thus, the effect of *Ddw1* on the plant height is more pronounced in spring triticale versus that in winter triticale, and it is similar to the effect of *Ddw1* in diploid rye where *Ddw1* can decrease the plant height by as much as 40% [16,18]. The differences in the effect of *Ddw1* on plant height depending on the habit type (spring or winter) may be associated with the differences in the environmental conditions under which plants pass from the vegetative to the generative phase. It also may be due to the interaction with developmental genes such as *Ppd* and *Vrn* that control this transition, as it was shown for *Rht* [29,30,31]. In general, the average volumes of agronomic traits of plants in the field experiment in 2017 notably differed from that in 2018 in both genotypes, *Ddw1Ddw1* and *ddw1ddw1*. This apparently could be due to average temperatures and different amounts of precipitation, especially in May–June when the generative organs are formed (Table 5). The environmental conditions in 2018 were more favorable for rapid development and maturation of spring triticale than in 2017, which in turn caused a difference over the years in other studied indicators.

The *Ddw1* allele is usually considered dominant with regard to rye height [16,32]. In the current study, the effect of *Ddw1* corresponds to incomplete dominance, i.e., the height of the heterozygous plants significantly differs from that of the homozygous plants. Partial dominant effect of *Ddw1* on the plant height has been described in winter triticale [20].

A decrease in absolute internode length induced by *Ddw1* was more pronounced in the peduncle internode and second upper internode; in the greenhouse F_2:3_ experiment, the relative decrease was more pronounced in the peduncle internode and first lower internode; and in the field F_4:5_ experiment, the decrease was more pronounced in the peduncle internode and second lower internode. The experiments in the primary octaploid triticale demonstrated a decrease in the peduncle internode (50.3%), second upper internode (17.3%), and third and fourth upper internode (21.9%) [27]. Thus, relative decrease in the internode length significantly depends on conditions of the experiment and other genes of the genotype. The environmental conditions, including temperature and water supply as well as developmental genes, apparently may influence the effect of dwarfing genes not only on plant height but also on productivity [29,30].

In our studies, *Ddw1* decreases the length of the internodes in a proportional manner. This phenomenon was not observed in the case of other dwarfing genes. For example, Rebetzke et al. [33] demonstrated that a decrease in the peduncle internode length was not proportional in the case of the GA-responsive *Rht13* gene that moderately correlated with increased spike and grain number. There may be space for further studies on the interaction between different GA-responsive genes in triticale to affect peduncle length and thus to increase plant productivity by increasing the availability of assimilates to the spike.

The data of the literature indicate that *Ddw1* increases the number of flowers and spike length in rye [16]. In our experiments, the number of spikelets was increased by 1.5 (5.9%) only in F_3:4_. However, spike length was decreased by 0.1–0.24 cm in the field F_3:4_ and F_4:5_ experiments, resulting in higher spike density. Thus, the effect of *Ddw1* on spike length occurs in opposite direction in spring triticale versus rye. In spring triticale, *Ddw1* increases the spike density due to a decrease in spike length in all experiments. The effect can be considered favorable in the case of a rainy summer during ripening because the fraction of sprouting grains is lower in the denser spikes apparently due to a decrease in moisture access to the grains [34]. However, additional studies of association of *Ddw1* with resistance to sprouting are required. 

Presence of *Ddw1* in the rye genotype increases the number of grains in a spike [16]. In our experiments, the number of grains in a spike did not change in the greenhouse and field experiments. However, grain weight per spike was decreased by 35% and 14–19% in the greenhouse and field experiments, respectively, in the case of *Ddw1*-positive genotype; 1000-grain weight was decreased by 30% and 15–22% in the greenhouse and field experiments, respectively. According to Kalih et al. [26], the presence of *Ddw1* in the hexaploid triticale decreased crop yield by 0.43 tons/ha and explained 10.4% of genotype variability of the grain yield. In our experiments, presence of *Ddw1* explained 9%, 13%, and 8% genotype variability of grain weight per spike in F_2:3_, F_3:4_, and F_4:5_, respectively. Additionally, Kurkiev [35] noted that *Ddw1*-harboring accessions of hexaploid triticale have lower productivity. 

In our experiments, the flowering and heading of the hexaploid triticale containing *Ddw1* was delayed by 3–9 days and by 4 days, respectively, versus that of the *Ddw1*-free plants. In the field experiments of Kalih (2015), plants with *Ddw1* headed by 2.2 days later on average, and Wolski et al. [26] also noted that the secondary *Ddw1*-containing triticale cultivars developed by them had a delay in heading.

The *Ddw1* gene is gibberellin-sensitive and similar to other gibberellin-responsive dwarfing wheat genes *Rht5*, *Rht12*, and *Rht 1*, significantly lowering the height of the plants (up to 40%); however, gibberellin-insensitive genes lower the height of the plants only by 10–12% [33,36,37]. *Ddw1* is considered an ortholog of the wheat dwarfing GA-responsive *Rht12* gene. The data of the literature indicate that *Rht12* significantly lowers the height of the plants (up to 46%) and does not influence the spike length while increasing fertility of the spikelets and delaying the heading by approximately 6 days [38]. Rebetzke et al. [37] reported that *Rht12* increased the grain yield and grain number and enhanced resistance to the lodging and harvest index while decreasing leaf size and grain weight. Thus, a significant decrease of height induced by the GA-responsive orthologous genes *Rht12* and *Ddw1* is associated with delayed heading and flowering and decreased grain size. Regression analysis of the data of the F_3:4_ and F_4:5_ field experiments indicates that the time of flowering and heading has medium strength significant correlation with the weight of 1000 grains, which is in agreement with the data of other authors obtained in wheat [37,39]. Thus, the negative effect of *Ddw1* on the developmental timeline should be compensated by coupling *Ddw1* with the *Ppd* and *Vrn* alleles that can accelerate the ripening [36]. In turn, this coupling may increase the 1000-grain weight. This approach may be particularly important in the Non-Black Earth region of Russia because delayed harvest can coincide with the rainy season resulting in considerable sprouting of the crops.

The ideotype of a variety for a particular agricultural zone should include adequate plant height and heading/flowering time that are influenced by multiple genes and can be efficiently developed based on the chromosome engineering and application of molecular markers [40,41,42]. *Ddw1* can be recommended as a major factor to decrease plant height in the spring triticale. *Ddw1* has a number of negative effects including delayed heading and flowering and smaller grain size. These drawbacks may be compensated by introducing other developmental genes. Additionally, dwarf plants are resistant to lodging that enables an increase in planting density and number of upright plants, thus compensating for a decrease in the yield of grain weight per spike.

## 4. Materials and Methods 

### 4.1. Plant Material 

Parental varieties of triticale carried the contrast combinations of the short stem alleles of rye including the winter triticale ‘Hongor’ (*Ddw1Ddw1* genotype; Krasnodar Research Institute of Agriculture named after P. Lukyanenko, Russia) and the spring triticale cultivar ‘Dublet’ (*ddw1ddw1* genotype; Danko Hodowla Roślin Sp. z o.o., Poland). ‘Hongor’ was used as the maternal plant; the F_1_ plants were hybridized and grown in 2015 in a greenhouse at the Center of Molecular Biotechnology, Russian State Agrarian University—Moscow Timiryazev Agricultural Academy, Moscow, Russia. The seeds of the maternal winter triticale ‘Hongor’ were seeded into the vegetation pots at 10 seeds per pot. During the tillering phase, the plants were placed into a spring chamber for 2 months. After the emergence during the heading phase, the anthers in a maternal plant were removed manually with fine forceps without damaging the stigma, and the spikes were isolated with butter paper bags. The maternal plants were pollinated by placing a spike of a cut flowering paternal plant under the paper bag with its stem placed in a bottle of water to keep it alive as long as possible. 

### 4.2. Greenhouse Experiment

The hybrid F_1_ plants were grown in the vegetation pots in 2016 in a greenhouse at the Center of Molecular Biotechnology. The F_2_ seeds were planted into vegetation pots at 10 seeds per pot. The F_2_ plants were grown under identical lighting with dosed watering and fertilization. Since ‘Hongor’ (paternal plant) is a winter triticale cultivar, a few winter plants were segregated in the F_2_ population and they were discarded based on their phenotype. A total of 136 F_2_ plants were analyzed, including 24 *Ddw1Ddw1* plants, 68 *Ddw1ddw1* plants, and 44 *ddw1ddw1* plants.

### 4.3. Field Experiment

The F_3_ seeds of spike of each of the F_2_ plants were manually threshed and combined into a single family. The homozygous F_3_ plants with the *Ddw1* and *ddw1* alleles were selected by genotyping and grown to produce the F_4_ plants. A total of 13 *Ddw1* homozygous and 9 *ddw1* homozygous F_3_ families were analyzed. The samples were withdrawn from each family during vegetation and thus, a total of 38 *Ddw1* homozygous and 34 *ddw1* homozygous F_4_ families were planted in the Field Station of the Timiryazev Agricultural Academy (55°50′ N, 37°33′ E) on 4 May 2017, and on 5 May 2018, using a breeding cassette drill SKS-6-10 with the following parameters: length of plot 1 m; width of plot, 90 cm; width between the rows, 30 cm; and distance between the plots, 50 cm. The weeds were manually removed and the plots were treated with pesticides to protect the plants from the pests. Each plant was manually harvested after complete ripening by August 27 in 2017 and August 18 in 2018. Threshing was performed using a spike thresher MKS-1M (MZOK Company, Moscow, Russia). The weather conditions in 2017 and 2018 are displayed in Table 5.

### 4.4. Phenotyping

The following phenotype features of each plant were recorded (all spike parameters were measured in the main spike): plant height (PH, cm), spike length (SL, cm), spikelet number per spike (SNS), spike density (SD, calculated as tenfold SNS divided by SL), grain weight per spike (GWS, g), grain number per spike (GNS), grain weight per spikelet (GNSpl, g), 1000-grain weight (TGW, calculated as thousandfold GWS divided by GNS, g), number of fertile tillers (NFT), number of internodes (NI), heading date (HD, days after sowing), flowering date (FD, days after sowing), harvest index (HI), peduncle length (first internode below the spike, cm), 2nd upper internode length (cm), 3rd upper internode length (cm), 2nd lower internode length, and 1st lower internode (the lowest) length (cm). Harvest index was calculated as a ratio of the grain weight from the main spike to the total weight of the unthreshed spike and the peduncle. Flowering and heading phases were determined visually for the whole family. The seeds were counted by the Seed Counter application [43].

### 4.5. Molecular Analysis

During the tilling phase, a leaf fragment of approximately 2 cm long was harvested from each plant and DNA was isolated by the CTAB method [44] for verification of the *Ddw1* hybrid genotype. DNA was extracted from each F_2_ plant to determine the alleles of the *Ddw1* genes. This information was additionally validated using selected F_3_ plants. The primers targeted the microsatellite locus REMS1218, which is tightly linked with the *Ddw1* gene [45]. The alleles of the REMS1218 microsatellites were assayed by PCR and subsequent fragment analysis was performed by a 3130xl Genetic Analyzer (Applied Biosystems, Foster City, CA, USA).

### 4.6. Statistical Analysis 

Mean, standard deviation, and min-max range were calculated for each phenotype. ANOVA was used to determine significance of differences according to the Fisher’s least significance difference (LSD) test by the Statistica 8.0 software (StatSoft) at 95% confidence level. The contribution of the number of internodes and the length of each internode into total reduction of plant height (Table 1), and the contribution of the allelic state of *Ddw1* and family into genotypic variation of the agronomic traits (Table 4) were estimated by the calculation of the correlation coefficients by the pairwise linear regression analysis. The ratios of internodes (see Table 2) were calculated as the length of a particular internode divided by the total plant height and expressed in percentages; additionally, since the plant height includes the spike length and in some plants may have an additional internode, the sum of the ratios is not equal to 100%.

The absolute effect of *Ddw1* was calculated as the difference between the mean phenotype feature in a group of plants with genotypes *Ddw1Ddw1* and *ddw1ddw1*; the relative effect was calculated as a ratio of the difference to the mean phenotype feature in a given group of plants with the *ddw1ddw1* genotype and expressed in percentages:

$$Effect\left(\%\right)=\frac{Mean\left(Ddw1Ddw1\right)-Mean\left(ddw1ddw1\right)}{Mean\left(ddw1ddw1\right)}100$$

## Figures and Tables

**Table 1:** Coefficients of regression between the length and number of internodes and plant height in various genotypes in the greenhouse and field experiments.

Stem Parameter	F_2:3_ (2014)				F_4:5_ (2018)		
	All Plants	*ddw1ddw1*	*Ddw1ddw1*	*Ddw1Ddw1*	All Plants	*ddw1ddw1*	*Ddw1ddw1*
Number of internodes	0.42 *	0.35 *	0.50 *	0.63 *	0.22 *	0.25 *	0.25 *
Peduncle	0.85 *	0.67 *	0.68 *	0.73 *	0.84 *	0.72 *	0.61 *
2nd UIN	0.88 *	0.80 *	0.73 *	0.60 *	0.82 *	0.57 *	0.59 *
3rd UIN	0.62 *	0.45 *	0.46 *	0.31	0.83 *	0.62 *	0.60 *
2nd LIN	0.68 *	0.57 *	0.48 *	0.27	0.68 *	0.37 *	0.35 *
1st LIN	0.33 *	0.23	0.05	−0.06	0.21 *	0.02	0.06

UIN, upper internode; LIN, low internode. Significant correlations (*p* = 0.05) are marked with asterisks (*).

**Table 2:** Ratios of the internode length in the spring triticale ‘Hongor’ × ‘Dublet’ population F_2:3_ in the greenhouse and families F_4:5_ in the field experiments.

Internode	F_2:3_			F_4:5_	
	*ddw1ddw1*	*Ddw1ddw1*	*Ddw1Ddw1*	*ddw1ddw1*	*Ddw1Ddw1*
Peduncle length	34%	32%	32%	36%	33%
2nd UIN	22%	22%	24%	24%	24%
3rd UIN	14%	16%	17%	14%	14%
2nd LIN	11%	12%	12%	9%	9%
1st LIN	6%	5%	6%	4%	4%

UIN, upper internode; LIN, low internode.

**Table 3:** Vegetative weight and harvest index in the spring triticale ‘Hongor’ × ‘Dublet’ family F_4:5_ in the field experiment.

Agronomic Trait	Genotype	
	*ddw1ddw1*	*Ddw1Ddw1*
Vegetative stem weight (LSD_0__.05_ = 0.03)	1.0 ± 0.3a	0.6 ± 0.2b
Vegetative spike weight (LSD_0__.05_ = 0.1)	2.8 ± 0.8a	2.4 ± 0.6b
Harvest index (LSD_0__.05_ = 0.01)	0.56 ± 0.07a	0.59 ± 0.07b

The letters demonstrate if the means are different (*p* = 0.05).

**Table 4:** Coefficients of regression (r) between *Ddw1* allelic state, family, and agronomic traits in the spring triticale ‘Hongor’ × ‘Dublet’ families F_3:4_ and F_4:5_ in the field experiment.

Agronomic Traits	F_3:4_		F_4:5_	
	Family	*Ddw1*	Family	*Ddw1*
PH	−0.63 *	−0.77 *	−0.18 *	−0.81 *
SL	−0.05	−0.03	−0.08 *	−0.10 *
SNS	0.28 *	0.25 *	0.00	−0.03
SD	0.43 *	0.34 *	0.11 *	0.08 *
GWS	−0.24 *	−0.36 *	−0.09 *	−0.28 *
GNS	0.14 *	0.10	−0.06	−0.05
GNSpl	−0.02	−0.04	−0.06	−0.04
1000GW	−0.54 *	−0.65 *	−0.08 *	−0.41 *
NFT	0.02	−0.07	−0.05	−0.06
NI	0.09	0.03	0.03	−0.10 *
HD	0.45 *	0.52 *	0.00	0.36 *
FD	0.42 *	0.42 *	0.00	0.33 *

Significant correlations (*p* = 0.05) are marked with asterisks (*).

**Table 5:** Weather conditions (sum of precipitation and average temperature) in 4 May–27 August 2017, and 5 May–18 August 2018.

Month	Sum of Precipitation		Average Temperature (°C)	
	2017	2018	2017	2018
May	162	105	10.3	16.1
June	266	107	14.4	17.3
July	221	190	17.9	20.5
August	41	39	19.9	20.3

## Supplementary Materials

The following are available online at https://www.mdpi.com/2223-7747/8/5/131/s1, Table S1: Effects of *Ddw1* in the spring triticale ‘Hongor’ × ‘Dublet’ population F_2:3_ in the greenhouse and families F_3:4_ and F_4:5_ in the field experiments.
