# Supplementary material for: Effect of Dwarfing Gene Ddw1 on Height and Agronomic Traits in Spring Triticale in Greenhouse and Field Experiments in a Non-Black Earth Region of Russia
Source: Plants (Basel). 2019 May 16;8(5):131. doi: 10.3390/plants8050131 (PMC6571949; doi:10.3390/plants8050131)
Supplement: Supplementary file 1 [file plants-08-00131-s001.pdf]

Table S1. Effects of *Ddw1* in the spring triticale 'Hongor' × 'Dublet' population $F_{2:3}$ in the greenhouse and families $F_{3:4}$ and $F_{4:5}$ in the field experiments.

Agronomic trait	Statistical parameter	$F_{2:3}$				$F_{3:4}$			$F_{4:5}$		
		ddw1ddw1	*Ddw1ddw1*	*Ddw1Ddw1*	Difference (%)	*ddw1ddw1*	*Ddw1Ddw1*	Difference (%)	*ddw1ddw1*	*Ddw1Ddw1*	Difference (%)
Plant height, cm	Mean	112.1a	78.8b	70.6c	**−41.5* (−37.0%)**	118.0a	84.6b	**−33.4* (−28.4%)**	83.4a	56.2b	**−27.2* (−32.6%)**
	SD	17.8	16.5	11.4	LSD$_{0.05}$ = 7.4	16.4	11.5	LSD$_{0.05}$ = 2.9	11.0	6.7	LSD$_{0.05}$ = 1.2
	Range	72.5–147	44–121	49–94		79–158	50–109		52.5–120	34–74.5	
Spike length, cm	Mean	10.5a	10.7a	10.2a	−0.3 (−2.9%)	10.3a	10.2a	−0.1 (−0.7%)	8.90a	8.65b	**−0.24* (−2.2%)**
	SD	4.1	3.2	2.7	LSD$_{0.05}$ = 1.8	1.4	1.1	LSD$_{0.05}$ = 0.3	1.3	1.0	LSD$_{0.05}$ = 0.15
	Range	4–3.5	6.5–25.0	5.5–19.5		7.5–17.0	6.7–13.5		4.5–13	6–12	
Spikelet number per spike	Mean	21.8a	21.0a	21.2a	−0.6 (−2.8%)	24.8b	26.3a	**1.5* (5.9%)**	22.8a	22.7a	−0.1 (−0.4%)
	SD	5	3.9	6.4	LSD$_{0.05}$ = 2.5	3.3	2.5	LSD$_{0.05}$ = 0.6	3.0	2.6	LSD$_{0.05}$ = 0.4
	Range	5–30	6–27	3–31		17–32	19–34		11–31	12–29	
Spike density	Mean	22.3a	21.4a	21.9a	−0.4 (−1.8%)	24.2b	25.8a	**1.6* (6.5%)**	25.9b	26.3a	**0.4* (1.7%)**
	SD	9.3	6.2	9.9	LSD$_{0.05}$ = 4.3	2.4	1.9	LSD$_{0.05}$ = 0.05	28.8	25.0	LSD$_{0.05}$ = 0.35
	Range	5.9–56.0	7.7–33.9	3.2–50.9		15.9–32.0	21.5–30.5		16.7–33.8	20.0–34.3	
Grain weight per spike	Mean	2.0a	1.5b	1.3b	**−0.7* (−35%)**	3.9a	3.2b	**−0.7* (−18.8%)**	2.1a	1.8b	**−0.3* (−14.3%)**
	SD	1	0.8	0.7	LSD$_{0.05}$ = 0.4	1.1	0.8	LSD$_{0.05}$ = 0.2	0.7	0.5	LSD$_{0.05}$ = 0.1
	Range	0.04–3.7	0.1–3.3	0.1–2.5		1.9–6.6	1.6–5.6		1.0–5.0	1.0–3.8	
Grain number per spike	Mean	48.8a	44.9a	43.7a	−5.1 (−10.5%)	68.3a	71.2a	2.9 (4.2%)	49.6a	48.3a	−1.3 (−2.6%)
	SD	18.2	15.3	15.2	LSD$_{0.05}$ = 8.5	16.3	12.6	LSD$_{0.05}$ = 3.0	12.0	10.1	LSD$_{0.05}$ = 1.4
	Range	6–80	10–72	3–65		34–110	42–113		22–97	23–80	
Grain number per spikelet	Mean	2.2a	2.1a	2.1a	−0.1 (−4.5%)	2.7a	2.7a	−0.04 (−1.4%)	2.17a	2.13a	−0.04 (−4.5%)
	SD	0.5	0.6	0.8	LSD$_{0.05}$ = 0.3	0.5	0.4	LSD$_{0.05}$ = 0.10	0.4	0.4	LSD$_{0.05}$ = 0.05
	Range	1.0–3.5	0.5–4.3	0.8–5.3		1.4–4.2	1.6–4.0		1.0–3.7	1.2–3.3	
1000−grain weight	Mean	39.2a	32.8b	27.4b	**−11.8* (−30.1%)**	56.7a	44.4b	**−12.3* (−21.8%)**	42.9a	36.6b	**−6.3* (−14.7%)**
	SD	14.3	13	12.2	LSD$_{0.05}$ = 6.9	7.7	6.7	LSD$_{0.05}$ = 1.5	7.3	6.1	LSD$_{0.05}$ = 0.9
	Range	6.7–60.0	7.1–60.0	2.8–50.0		33.3–72.4	25.1–59.1		19.3–61.8	21.6–58.1	
Number of fertile tiller	Mean	2.0a	1.9a	1.9a	−0.1 (−5%)	3.2a	3.0a	−0.2 (−7.3%)	2.30a	2.17a	−0.13 (−4.3%)
	SD	0.8	0.8	1	LSD$_{0.05}$ = 0.4	1.8	1.6	LSD$_{0.05}$ = 0.4	1.1	1.0	LSD$_{0.05}$ = 0.13
	Range	1–4	1–3	1–5		1–10	1–11		1–6	1–6	
Number of internodes	Mean	5.16a	5.05a	4.88b	**−0.28* (−5.8%)**	5.01a	5.06a	0.05 (0.9%)	5.20a	5.09b	**−0.11* (−1.9%)**
	SD	0.43	0.47	0.45	LSD$_{0.05}$ = 0.23	0.8	0.7	LSD$_{0.05}$ = 0.16	0.5	0.6	LSD$_{0.05}$ = 0.07
	Range	4–6	4–6	4–6		3–8	2–6		4–7	3–6	
Heading time (days after sowing)	Mean	41.1a	41.5a	41.6a	0.5 (1.2%)	64.2b	73.1a	**8.9* (13.9%)**	54.6b	57.7a	**3.1* (5.7%)**
	SD	4	5	3.9	LSD$_{0.05}$ = 2.3	2.8	3.1	LSD$_{0.05}$ = 0.8	3.4	5.7	LSD$_{0.05}$ = 0.6
	Range	34–49	13–51	35–48		61–71	70–78		48–72	53–72	
Flowering time (days after sowing)	Mean	50.6a	50.3a	50.4a	−0.2 (−0.4%)	76.9b	81.1a	**4.2* (5.5%)**	57.3b	61.1a	**3.8* (6.6%)**
	SD	3.0	3.1	3.2	LSD$_{0.05}$ = 1.7	2.3	3.1	LSD$_{0.05}$ = 0.7	3.7	6.1	LSD$_{0.05}$ = 0.6
	Range	44–55	45–55	46–55		73–81	78–86		53–76	55–76	

Peduncle length	Mean	38.4a	24.9b	22.8b	**−15.6* (−40.6%)**	30.1a	18.6b	**−11.5* (−38.2%)**
	SD	7.7	5.8	3.7	LSD$_{0.05}$ = 3.1	7.8	4.0	LSD$_{0.05}$ = 0.8
	Range	21–56	8–42	15–29		7.5–82.5	5.5–31	
2nd upper internode length	Mean	24.9a	17.5b	16.6b	**−8.3* (−33.3%)**	19.7a	13.2b	**−6.5* (−33.0%)**
	SD	4.2	3.6	2.6	LSD$_{0.05}$ = 2.0	3.2	2.5	LSD$_{0.05}$ = 0.4
	Range	15–31	10–26	11–21		7.5–29.5	8–20.5	
3rd upper internode length	Mean	15.9a	12.6b	12.1b	**−3.8* (−23.9%)**	11.7a	7.8b	**−3.9* (−33.3%)**
	SD	3.8	2.6	1.7	LSD$_{0.05}$ = 1.5	2.0	1.6	LSD$_{0.05}$ = 0.2
	Range	7–30	4.5–18.5	9.5–15.5		5–18.5	0.5–14	
2nd lower internode length	Mean	12.3	9.4	8.6	**−3.7* (−30.1%)**	7.5a	4.8b	**−2.7* (−36.0%)**
	SD	2.8a	2.0b	2.2b	LSD$_{0.05}$ = 1.2	1.8	1.2	LSD$_{0.05}$ = 0.2
	Range	5.5–19.0	5.0–15.5	4.5–13.0		1.0–16.5	1.5–11.5	
1st lower internode length	Mean	6.3a	4.1b	4.1b	**−2.2* (−34.9%)**	3.5a	2.5b	**−1.0* (−28.6%)**
	SD	3.3	2.6	2.3	LSD$_{0.05}$ = 1.4	2.1	1.7	LSD$_{0.05}$ = 0.3
	Range	2–15	0.5–11	1–8		0.5–25	0.5–25	

Significant differences (p = 0.05) are marked with asterisks (*) and typed in bold; the letters demonstrate if the means are different.
